# Practical Chemistry of the First Year in the Medical Department of the University of Texas

**Published:** 1899-12

**Authors:** 


Practical Chemistry of the First Year in the Medical Department of the University of Texas. By S. M. Morris, B. S., M. D., Professor of Chemistry and Toxicology, Medical Department of the University of Texas. Second edition. Revised and Enlarged.

This little book lends special interest to the medical men of Texas on account of its authorship. Professor Morris is a native Texan and a product of the University, a man who has won his way by merit, and is a favorite with the faculty and the students.

The work is intended as a laboratory guide, including the exercises in inorganic chemistry as taught by Professor Morris to first year students, and is a companion work to "Practical Chemistry, Including the Exercises in Medical and Pharmaceutical Chemistry," adapted to second year students (the latter work has separate notice). Every other page is left blank for notes in the course of the year's work. Nearly the entire book is devoted to laboratory experiments, there being about two hundred in all. As a thoroughly practical guide for students there seems to be nothing lacking. B.

